# Developing Best Practices in Co‐Production With Lived Experience Experts in Bipolar Research: Insights From the AMBIENT‐BD Lived Experience Advisory Panel

**DOI:** 10.1111/hex.70887

**Published:** 2026-10-05

**Authors:** Raahat Manrai, Amber Roguski, Amy Ferguson, Iain Campbell, Daniel J. Smith, Aja L. Murray, Maria Gardani

**Affiliations:** ^1^ Division of Psychiatry, Centre for Clinical Brain Sciences University of Edinburgh Edinburgh Scotland UK; ^2^ School of Psychology, Philosophy and Linguistic Science University of Edinburgh Edinburgh Scotland UK; ^3^ School of Health in Social Science University of Edinburgh Edinburgh Scotland UK

**Keywords:** bipolar disorder, consultation, co‐production, engagement, lived experience, participation

## Abstract

**Introduction:**

Co‐production is becoming an established methodology in mental health research, yet there remains a lack of condition‐specific frameworks to guide its design and implementation. For bipolar disorder in particular, there is limited evidence on how to meaningfully engage lived experience experts in the context of possible mood instability and other impairments. This paper presents insights from a consultation with people with lived experience of bipolar disorder, aimed at developing recommendations for conducting co‐production with people with bipolar.

**Methods:**

A one‐off, 1‐h online consultation was conducted with four members of the AMBIENT‐BD Lived Experience Advisory Panel (LEAP). The consultation used a semi‐structured focus group format, guided by a topic guide, to explore strategies for recruiting and engaging co‐producers in bipolar disorder research. Data were analysed using reflexive thematic analysis.

**Results:**

Four overarching themes were developed: (i) *Communicating with co‐producers‐* capturing how trusted channels and regular contact support recruitment and sustained engagement; (ii) *Providing accessible information*‐ addressing the need to balance sufficient detail with readable, low‐burden formats during periods of instability; (iii) *Building and sustaining trust*‐ highlighting trust in the research process and partnerships with third‐sector organisations as preconditions for meaningful involvement; and (iv) *Perceived impact as a motivator*‐ reflecting how community belonging and tangible incentives shape willingness to participate. A provisional set of practical recommendations was derived from these themes, including recruiting through trusted organisations, using non‐technical language and multiple formats, offering meaningful incentives, and using low‐burden communication methods during periods of unwellness.

**Conclusion:**

Grounded in lived experience perspectives, this paper conceptualises co‐production as a relational process in which trust, accessible communication and visible impact form the foundation for meaningful engagement with people with bipolar disorder. Positioned as a consultative contribution on the IAP2 Spectrum of Public Participation, the paper offers practical, mental health‐specific guidance for researchers seeking to develop advisory groups and other participatory approaches with the bipolar population.

**Patient/Public Contribution:**

This study has been conducted in partnership with people with lived experience of bipolar disorder. They were involved in the conceptualisation, design and data collection of the study. They helped to inform the process of conducting a co‐production with people with bipolar disorder. Findings from the consultation were shared with them in a separate feedback session.

## 1 Introduction

Co‐production of research evidence, policy and practice is becoming increasingly prevalent within mental health research [1, 2]. Traditional biomedical and clinical research has generated important advances for people living with mental health conditions, including improvements in diagnosis, pharmacological treatment and psychosocial intervention. Similarly, there is growing recognition that research conducted primarily *on* rather than *with* people can overlook priorities that matter most to those with lived experience. This could lead to misrepresentation of community needs or produce findings that are less feasible in their everyday life [3, 4]. Therefore, there is growing emphasis on including lived experience within research decision‐making, along with recognising the challenges that come with including experts by experience within research [1, 5].

However, participatory approaches in mental health remain unevenly developed across conditions and study designs. A recent scoping review of using co‐production study designs in sleep research highlighted the lack of clarity and a clear framework for participatory designs in research [6]. This paper focuses on informing the design of co‐production within bipolar disorder research, drawing on consultation with lived experience experts already involved in the AMBIENT‐BD project.

### 1.1 Including Lived Experience Involvement in Bipolar Research

Bipolar disorder is a severe mental illness characterised by recurrent episodes of mania, hypomania and depression, additionally accompanied by sleep and circadian disruption and functional impairment [7]. It is marked by periods of fluctuating wellness affecting concentration, energy, confidence and capacity to engage with research [3]. These factors could create distinct challenges for participatory research where people may disengage during a mood episode or experience institutional mistrust due to experiences of stigmatisation or impersonal recruitment strategies.

Therefore, lived experience involvement is important for bipolar disorder research. Previous priority‐setting and community‐based work suggest that people with bipolar disorder often value outcomes beyond symptom reduction, improving quality of life, self‐management during episodes and reducing stigma in everyday life [3, 8]. People with lived experience can provide practical insight into research processes, study methods and research materials such as participant information sheets, recruitment strategies and dissemination resources [7]. Researchers might risk designing co‐production processes that are tokenistic, burdensome, or inattentive to the power imbalance between researchers and community members [9].

There is an increasing amount of meaningful literature on using participatory approaches in bipolar research. The Collaborative RESearch Team to study psychosocial issues in bipolar disorder (CREST.BD) had demonstrated how meaningful partnerships between researchers and people with bipolar disorder can shape research priorities, methods and translation of knowledge [3, 8]. Similarly, other participatory initiatives, including youth‐focused CBPR projects, show the value of embedding community partners across all stages of research process. However, this literature has focused on involving people with bipolar disorder *within* research projects, rather than consulting with them on *how* to design co‐production and feasible advisory processes. There is also a limitation on condition‐specific practical guidance for recruiting and sustaining advisory groups in the context of mood instability.

#### 1.1.1 Existing Frameworks and the Need for Bipolar‐Specific Guidance

Despite growing emphasis on co‐producing research with people with lived experience, researchers often lack practical, condition‐specific frameworks to guide and implement research. The INVOLVE guidance sets out principles for authentic co‐production and highlights challenges related to power, flexibility and evaluation [10]. Smith, Williams [2] emphasise that having a shared resource helps define roles, structure processes and clarify the relevance of participatory research within specific contexts. Community‐based frameworks similar to those included in CREST.BD rely on equitable partnership, priority settings and integrated transfer of knowledge [3, 8]. The IAP2 Spectrum helps researchers communicate the level of influence being offered to community partners [11].

While these frameworks are valuable, they are largely generic or oriented towards long‐term research partnerships rather than implementing and operationalising research in terms of research processes. Without bipolar‐specific guidance, co‐production efforts risk reproducing existing power imbalances, overburdening people during periods of unwellness, relying on inaccessible language or recruiting those already embedded in research networks [12, 13]. There is a notable absence of practical guidance on how to meaningfully engage people whose condition may involve fluctuating mood levels, reduced confidence in clinical research, and experiences of disenfranchisement. This can reduce researchers’ ability to conduct rigorous and ethical co‐production that centres the voices and expertise of people with bipolar disorder.

Therefore, this paper aims to inform the development of structured, practical recommendations for co‐production while addressing a gap often overlooked in previous studies, especially around how engagement with bipolar disorder should be designed. It also aims to understand specific needs and preferences within the bipolar disorder community relating to participation in different types of research studies.

### 1.2 Types of Co‐Production and Levels of Engagement

Co‐production methodology forms part of the broader umbrella of participatory approaches in applied health research to highlight partnership between communities with lived experience and academic institutions. Oliver, Kothari [14] define co‐production as a collaborative research practice in which researchers interact with multiple stakeholders. Study designs include advisory groups or consultations, workshops and peer interviews, among others.

Approaches further vary in the level of involvement, and the stage at which lived experience experts are engaged [4], and degree and nature of involvement exist on a spectrum [9]. Advisory groups typically provide ongoing guidance throughout the research process, from conceptualisation to dissemination. Workshops offer structured opportunities for collaborative input on specific areas of study design or interpretation. Peer interviews leverage shared experience between the interviewer and participant to generate more contextual data.

The International Association for Public Participation (IAP2) Spectrum of Public Participation provides a widely used framework for clarifying the role of community partners in decision‐making, ranging from Inform, through Consult, Involve and Collaborate, to Empower [11]. On this spectrum, consultation involves seeking input at specific time points, whereas fuller partnership involves shared decision‐making and co‐leadership. The INVOLVE guidance similarly distinguishes co‐production from lower forms of involvement, defining co‐production as an approach in which researchers, practitioners and the community members share power and responsibility from conceptualisation to dissemination of a research project [10]. The principles of sharing power, including all perspectives, respecting and valuing knowledge, reciprocity, and building and maintaining relationships, offer useful standards against which co‐production processes can be assessed [9].

This paper reports a one‐off consultation about the co‐production itself, in which people with lived experience provided guidance on how to meaningfully engage the bipolar disorder community in shared decision‐making and co‐design. Within the IAP2 framework, it sits on the Consult level, while aiming to inform later stages of AMBIENT‐BD study that aim for deeper collaboration. It is important to be transparent about this process to ensure transparency and avoid overstating [4, 10].

#### 1.2.1 Importance of Meaningful Participation

Regardless of the specific approach, effective participatory research requires meaningful engagement that goes beyond tokenistic inclusion. This can help ensure that the contributions of people with lived experience shape research questions, methods and outcomes [5]. Navigating power sharing remains complex and requires careful attention to ensure collaboration is genuine rather than performative [9]. Participatory work within bipolar disorder research offers opportunity to develop meaningful relationships with the community and improving the relevance and acceptability of study design [7]. There remains a specific need to consult people with lived experience on how best to design meaningful engagement, shared decision‐making and co‐design approaches that are considered appropriate and feasible for the bipolar disorder community.

### 1.3 Present Paper

The current study is one of the first to directly include the voices of people with bipolar disorder in developing a framework for co‐production research. It presents findings from a one‐off advisory consultation with four members of the Lived Experience Advisory Panel (LEAP) established as part of the AMBIENT‐BD project [15]. This consultation aimed to gather insights and develop recommendations on how to effectively engage people with bipolar disorder in co‐producing research, specifically focusing on the design and implementation of advisory groups.

This work contributes to the limited but growing body of work on engaging lived experience experts (bipolar disorder in particular) in participatory research. The findings from the consultation aim to offer practical recommendations for future researchers seeking to develop advisory groups and other participatory approaches with this population [16]. It further highlights considerations specific to the needs and experiences of people living with bipolar disorder, along with generating a framework for conducting co‐production with people with bipolar disorder and other severe mental illness.

## 2 Methods

### 2.1 Context of the Study

The current study is part of the wider AMBIENT‐BD research project. AMBIENT‐BD is a Wellcome‐Trust funded research study aiming to use sleep and circadian rhythms to understand clinical trajectories and outcomes in bipolar disorder [15]. Co‐production is a core component of AMBIENT‐BD, and the project has a dedicated Lived Experience Advisory Panel (LEAP) made up of people with lived experience of bipolar disorder. The LEAP was established at the outset of the project to provide ongoing guidance and oversight throughout all stages of the research, including protocol development, participant recruitment, planning for data collection and dissemination strategies. LEAP members were recruited through Bipolar Scotland and word of mouth. The opportunity to participate in the current research regarding co‐production for bipolar research was communicated via email to all LEAP members. This study focuses on a comprehensive one‐off consultation with the members of the project's Lived Experience Advisory Panel (LEAP). The consultation reported in this paper was specifically designed to gather LEAP members’ insights on how to effectively recruit and engage additional people with bipolar disorder in future advisory groups and co‐production activities.

#### 2.1.1 Characteristics of AMBIENT‐BD LEAP Members

The AMBIENT‐BD LEAP consisted of four people with a bipolar disorder diagnosis (one male, three female). Both Type I and Type II bipolar diagnoses were represented within the group. The AMBIENT‐BD LEAP was established to disseminate and exchange knowledge collected in the wider AMBIENT‐BD research project. Specifically, the LEAP will be creating pieces of music using objective sleep data to depict lived experience of sleep in bipolar and has advised the workstreams of the project on the feasibility, acceptability and methodology of the study. The LEAP was recruited through collaboration with Bipolar Scotland and people with background in music composition and production. Some LEAP members were also involved in the conceptualisation and development of the AMBIENT‐BD research and grant application.

### 2.2 Ethical Considerations

The School of Health in Social Science Research Ethics Committee provided ethical approval for this study (Ref 23‐24CLPS036).

It was important to seek ethical oversight for this consultation. Despite being a participatory study, the decision to seek ethical approval for this consultation was considered appropriate for the following reasons. First, it provided formal recognition of LEAP members’ contributions and ensured their participation was documented and protected [17]. Second, it required the research team to articulate clear processes for consent, confidentiality, data management and potential risks or distress, which are important considerations, especially within the context of participatory research [18]. Third, ethical oversight helped maintain reflexivity about power dynamics and the potential for exploitation, ensuring that LEAP members’ time and expertise were appropriately valued [19].

### 2.3 Procedure

This consultation was an initial step of a broader co‐production process being conducted with people with lived experience of bipolar disorder. The purpose was to compile LEAP members’ recommendations that would inform the design of subsequent advisory groups with a wider group of people with lived experience of bipolar disorder. The consultation was held online using Microsoft Teams and lasted approximately 90 min. It was conducted as a semi‐structured focus group. Four LEAP members attended, together with the lead author (RM) and additional research team facilitators (A.R., A.F., and I.C.). Ground rules were established at the start to support the safety of the LEAP members. A designated safety officer from the research team was present, even though none of the LEAP members sought their help. All LEAP members provided written informed consent prior to the consultation. LEAP members were compensated at £25 per hour, in line with NIHR payment guidance for public contributors at the time of the study.

A semi‐structured topic guide was used to explore prior research experience, strategies for reaching diverse communities, ways to recruit co‐producers, communication preferences, information needs, trust and perceived impact. The topic guide was informed by existing co‐production and participatory research literature, including guidance on meaningful involvement, power sharing and accessible communication [2, 9, 10] and provided as File S1. The LEAP members were asked to review the research materials, such as participant information sheets and study recruitment materials, for the co‐production study. The consultation was audio‐recorded and transcribed verbatim. The transcript was anonymised during transcription by providing pseudonymous identifiers (LEAP C, M, N and T).

### 2.4 Analysis

The data were analysed using Reflexive Thematic Analysis [20]. The analysis used an inductive orientation, generating codes and themes from the data set rather than applying a pre‐existing coding framework. The analysis was underpinned by a critical realist ontology and a contextualist epistemology. This positioning recognises that LEAP members’ accounts refer to real experiences and conditions shaping research involvement, while also acknowledging that meaning is produced through language, social context and interpretative activity of the research [20].

Stage one included transcription and data familiarisation with the data through repeated reading and note‐taking. Stage two consisted of line‐by‐line coding across the data set, generating both semantic codes (close to participants’ explicit meanings) and latent codes (interpreting underlying assumptions and implications relevant to LEAP members). Stage three included collating codes into overarching themes. Themes were then reviews against coded extracts and the entire data set for coherence and distinctiveness in stage four. In stage five, themes were refined and named, and sub‐themes were identified. Finally, in stage six, the analysis was written up. Coding and initial theme development were led by R.M. Themes were then reviewed, discussed and supervised by A.L.M. and M.G.

### 2.5 Reflexive Positionality

The lead author (R.M.) is an early‐career academic researcher with no personal lived experience of bipolar disorder. She also had no prior relationship with the LEAP members before the consultation. This outsider position may have limited experiential insight into the emotional and practical realities of living with bipolar disorder, along with offering some analytic distance from insider assumptions. Due to R.M.'s outsider position, it was deemed necessary to hold this consultation prior to conducting the actual co‐production study.

R.M.'s positioning as an academic researcher under the umbrella of institutional authority is relevant to the power dynamics of the consultation. Although LEAP members were invited to advise on process design, she made final decisions over research questions, analysis and write‐up. Additionally, LEAP members were already engaged with AMBIENT‐BD research study, which may have shaped their reflections in relatively constructive directions and reduced the likelihood of more critical accounts from people less familiar with research.

## 3 Results: Effective Strategies to Include People With Bipolar Disorder Within Scientific Research

The consultation explored four areas of research participation: communication with potential and existing co‐producers; accessible information provision; trust between researchers and people with lived experience; and the motivational role of perceived impact. These themes are interrelated with each other and consistent with previous evidence from lived experience‐informed research [21]. The themes have been summarised in Table 1.

**Table 1:** Theme summary table.

Overarching theme	Sub‐theme(s)	Theme description
Communicating with co‐producers	Recruitment through trusted channels Negotiating frequency of contact and participant burden	Practical insights into reaching co‐producers and sustaining contact without overwhelming them.
Providing accessible information	Balancing detail, clarity and format	How information content and presentation shape decision to engage.
Building and maintaining trust	Trust in the research process Trusted organisations as intermediaries	Trust as a precondition for meaningful and sustained engagement.
Visible impact as a motivator	Belonging and community Incentives and direct benefits	How collective and personal benefits motivate involvement.

### 3.1 Communicating With Co‐Producers

This theme primarily focused on how to reach and recruit co‐producers from the bipolar disorder community. LEAP members focused on how people with bipolar disorder learn about research opportunities and how communication can support (or undermine) potential involvement. They further stated that communication with community partners should be treated as a relational practice to provide legitimacy, respect and care.

#### 3.1.1 Recruitment Through Trusted Channels

LEAP members emphasised that people with bipolar often learn about participation opportunities through trusted and guided channels rather than general advertising. They described convenience sampling methods via peer support groups and word of mouth as primary way of engaging with research because these channels came from trusted sources.

The reason that I know about this group was through the support group, Bipolar Scotland and I think that… speaking to people or word‐of‐mouth is a big thing.

LEAP (T)

This suggests that recruitment is not different from the process of trust building, where information is more likely to be well received when it is provided through relationships and organisations that are already considered safe.

#### 3.1.2 Negotiating Frequency of Contact and Participant Burden

LEAP members are advised to understand the balance between regular contact and the risk of overburdening co‐producers. During the consultation, ‘too much contact’ in terms of frequent emails or notifications was framed as feeling pestered or emotionally triggered. This could be attributed to the bipolar diagnosis itself, where people can become aversive during periods of low mood or experience anxiety and sensory overload due to perceived high levels of communication.

Keep in touch but don't push and don't be kind of be… overly… not be a pest, basically, you know, because that can trigger a lot of things as well.

LEAP (N)

If I got emailed too much, I would just block it no matter what. I don't even… I would feel like I am being sold to and I would probably block it.

LEAP (M)

LEAP members also emphasised that communication during the co‐production process should include sharing visible progress and updates on the research. This form of ongoing communication was seen as important for sustained engagement and improving transparency of the research process.

It helps that you share how the progress is going. What are you learning? How is the research going? How are they contributing? Share the little… little fruits along the way. It is important, I think!

LEAP (C)

Effective communication in bipolar disorder research includes calibrated contact, including connection and transparency, to manage communication with co‐producers.

### 3.2 Providing Accessible Information

This theme captures the tension between providing enough detail to set clear expectations and ensuring materials remain usable and accessible, particularly during fluctuating mood episodes. LEAP members indicated that the manner in which information is provided shapes decisions to engage, irrespective of the study design.

Following from the previous theme, the LEAP members emphasised that *how* information is provided is important in engaging people with bipolar disorder in research, regardless of the research design. The manner in which the information is provided helps to inform the decision to engage with co‐production opportunities. This theme further aims to capture the tension between providing sufficient detail to set clear expectations while ensuring materials remain accessible, particularly during fluctuating mood episodes.

I think make sure that you know… that people know how long the study is for, so, they know what to expect. Just basically for all of the details, just so they know what to expect so nothing is a surprise. Because if anything is a surprise, it can be quite off putting.

LEAP (N)

Providing information could also be extended to providing regular updates and/or organising a feedback session for the co‐producers. LEAP members stated that the visibility of potential impact could help reduce their frustration with the time taken in research processes.

Two sub‐themes emerge within this theme, which are discussed below. In the sub‐themes, LEAP members stated the distinction between determining the appropriate amount of information to share and ensuring that the information is presented in accessible and readable formats.

#### 3.2.1 Balancing Detail, Clarity and Format

LEAP members stressed the need to set clear expectations and avoid surprises, especially because research content and materials, and often includes vulnerable and sensitive topics. Providing information before sessions would help co‐producers make informed choices and respect their boundaries.

Make sure that you know… that people know how long the study is for, so they know what to expect. Just basically for all of the details, just so they know what to expect. Just basically for all of the details, just so they know what to expect so nothing is a surprise. Because if anything is a surprise, it can be quite off‐putting.

LEAP (N)

Another LEAP member stated that in‐depth detail is required to raise understanding of information and awareness about the study using simple (jargon‐free) language. They stated that scientific phrasing could be seen as a barrier rather than a marker of legitimacy. LEAP members further highlighted that during periods of poor mental health, reading dense text can be overwhelming and dissuade them from taking part in the study.

For me, the less scientific language, the better, but as [another LEAP member] said, you don't want people to be surprised, you want people to know what they are getting into.

LEAP (T)

LEAP members repeatedly suggested video formats as alternatives to written materials for consent forms and flyers. When asked to reflect and provide feedback on the recruitment flyer for the co‐production study during the consultation, LEAP members recommended simpler wording. They further recommended that the visual design, colour scheme and graphics used on the flyer mattered when informing people about the study.

The wording could be more straightforward and more succinct, because it is still a lot of text and a lot of information and wording like co‐production is still not very straightforward. If I didn't know about the study, I would have read the whole flyer and still don't know what it is trying to tell me.

LEAP (C)

Therefore, this theme suggests that information provided in the context of bipolar disorder research needs to understand the issues around accessibility, readability and safety. Providing clear information would help co‐producers to know what is expected and make informed decisions.

### 3.3 Building and Sustaining Trust

Trust permeated the consultation and was framed as a precondition for meaningful engagement within participatory research. LEAP members described trusted relationships with the bipolar disorder community for longer‐term involvement both within participatory and conventional research designs.

Trusted relationships can be established both directly and indirectly. Directly, researchers can spend time building rapport with community members at credible locations. LEAP members suggested that meeting consistently helps to gradually build a sense of safety and familiarity.

Every three months, a meeting in‐person, the same venue to build up that sense of trust, that sense of connection… and keeping it kind of… real.

LEAP (N)

This theme also helps to reflect on power differential between researchers and lived experience experts. LEAP members reflected about concerns of feeling like ‘guinea pig’ and the importance of verified organisations to recruit co‐producers, which highlighted an institutional inequality between researchers and community members. While this consultation was not designed as an analysis of power, trust‐building functions as a practical response to reducing that imbalance.

#### 3.3.1 Individual Trust in the Research Process

One LEAP member noted that trust is particularly important before advertising research to clinical populations, because people with severe mental illness may be mistrustful of research processes. This mistrust was described as potentially linked to experiences associated with bipolar disorder itself, including paranoia, rather than solely as a general attitude towards science.

With people with bipolar, I get paranoid… ‘What is going on? Is this real? Is this a scam? What is it?’ you know and I get really concerned… maybe to actually have an in‐person sort of… recruitment fair almost. So, it is actually a presentation with people, a specific location that is trustworthy, that people are going to believe and… it is legitimate, so you know… they are not scared, because they shouldn't be, because it is a legitimate thing.

LEAP (N)

This sub‐theme further suggests that trust in bipolar disorder research is both relational and clinically informed. Hence, it is advisable for researchers to design engagement practices that recognise how symptom experiences, prior institutional encounters and perceptions of research motives can shape willingness to take part.

#### 3.3.2 Trusted Organisations as Intermediaries

All LEAP members stressed working with trusted organisations to facilitate relationships and engagement with community partners.

Verified groups like Bipolar Scotland… reaching out to proper groups, doing talks… just brief kind of talks, maybe a Q&A afterwards, just to kind of get people engaged.

LEAP (N)

Trusted organisations and peer‐to‐peer support networks were consistently described as the primary channels through which LEAP members learned about research. Peer support groups were valued because trust would be built through shared experience over time.

The reason that I knew about it was through the support group, Bipolar Scotland and I think that… speaking to people or word‐of‐mouth is a big thing.

LEAP (T)

Peer‐to‐peer support groups are something that I always really enjoyed, and I went… I started when I wasn't so healthy. And they have seen me through example.

LEAP (M)

This finding closely aligns with principles of community‐based participatory research, where established community organisations assist to bridge the gap between academic institutions and community members [3]. In this consultation, trusted organisations brokered trust, along with acting as catalysts of recruitment.

### 3.4 Perceived Impact as a Motivator

This theme summarises how the perceived impact of research, especially on individuals’ lives and the wider community, facilitated as motivators to engage with research. They further describe both collective benefit and more immediate personal incentives as important factors in engagement. Generating impact also meant communicating the visibility and relevance of research.

A LEAP member described how dissemination through media and other opportunities could make involvement more meaningful.

That actually makes it more exciting to be a part… to be any part of it you know… to see that it is news worthy and that things are growing and… you know… going in a positive direction. I just thought that it was highly motivating to see those kinds of efforts. And I think for me that is a big motivator!

LEAP (M)

#### 3.4.1 Belonging and Community

LEAP members viewed belonging and contribution to the wider community as key motivators. The participatory design of co‐production was seen as potentially facilitating shared experience between community partners.

Feeling like you are contributing, feeling like you are a part of something, like a sense of belonging, sometimes helps too!

LEAP (T)

Taking part was also described as a way of helping others, reinforcing the communal value of research.

I am sure it will help a lot of people, once it is finished, it is all sorted out, so yeah. It is exciting to see what happens!

LEAP (N)

This further suggests that motivation for involvement could be both altruistic and self‐interested. Lived experience involvement could also be tied to identity, belonging and engaging for the wider community.

#### 3.4.2 Incentives and Direct Benefits

Beyond collective impact, LEAP members identified personal benefit as an important consideration. This included feeling that researchers were exploring beyond familiar treatment assumptions, as well as receiving something of concrete value from participation.

That will be another point of consideration and see how that affects my wellbeing so that would be an extra incentive. So, it has to be, there has to be some way that you can help the participants as well.

LEAP (C)

LEAP members further stressed that financial incentives were important for people with lived experience. They noted that financial incentives might assist people to feel renumerated if they struggle to earn consistently because of their mental health.

Just do what you are doing on this! You know… reassuring people that there is support there, if necessary and cash! I think it, I said that a while ago, money is a big… a big incentive, you know, so yeah!

LEAP (N)

This finding is further consistent with wider patient‐oriented research literature showing that fair payment, visible influence and reciprocal benefit are key supports for sustained partnership [13, 22]. Incentives were framed as important signals to demonstrate that lived experience expertise is valued.

## 4 Practical Recommendations for Co‐Producing Research With People With Bipolar Disorder

Drawing on the four themes developed from the consultation, a provisional set of recommendations is proposed for researchers planning co‐production with people with bipolar disorder. These recommendations are inductively derived from LEAP reflections within the AMBIENT‐BD context and can be adapted carefully for other settings and populations. Some of the recommendations were directly informed by the analysis, while others were informed by the consultation and existing literature.

− **Recruiting through trusted third sector organisations.** There is an important way to ensure meaningful representation within co‐production groups. LEAP members encouraged recruiting co‐producers through trusted organisations like Bipolar Scotland and Bipolar Edinburgh, which have trusted relationships with the bipolar disorder community. This approach leverages existing trust to facilitate meaningful engagement between co‐producers and the research team.
− **Using non‐technical language and multiple formats.** It is important to avoid jargon or technical terms like co‐production in recruitment materials, consent forms, and communications. Offer information through bullet points, short videos, brief verbal summaries and cohesive video or visual design.
− **Consent as a conversation instead of a fixed document.** The LEAP members highlighted that traditional written consent forms can present significant barriers to engagement, particularly during periods of poor mental health. It is recommended for researchers to treat consent as an ongoing conversation, allowing researchers to present information in an accessible manner. This conversational approach reinforces the collaborative nature of co‐production and ensures genuine comprehension rather than signing a consent form as a tick box exercise.
− **Recruiting participants from diverse backgrounds.** While recruiting people from the community is important, it is also important for researchers to recruit people from diverse backgrounds. This was suggested to avoid over‐consulting the same community partners for different research studies. It is therefore important for research studies for co‐producers to belong to diverse backgrounds.
− **Offering incentives and communicating direct benefits.** The LEAP members highlight the importance of incentives to meaningfully engage advisors in the co‐production process. These incentives could be financial or could be something of personal values‐ like summary of data about their own health, access to new measures or devices, knowledge relevant to self‐management of their mental health.
− **Sharing progress regularly and visibly.** Having a feedback loop is an important aspect to the co‐production process. The LEAP members stated the importance of providing regular updates on the process, celebrating milestones and recognising their contributions at different steps. This feedback loop could also lead to having a ‘celebratory session’ or a closing meeting to acknowledge the co‐producers’ contributions and efforts on the process.
− **Communication during periods of unwellness.** The LEAP members stated having regular check‐ins with the co‐producers without intervening in their daily lives. Having colour‐coded wellbeing indicators, for example, or simple ‘yes, no, maybe’ is helpful for people to express their moods without extensive written communication.
− **Respecting the boundaries of participation.** The LEAP members advised against assuming the inclusion of family members, partners or carers, people who otherwise are considered people with vicarious lived experience. Co‐producers or participants (in general) may experience fears of burdening their family members or carers, and the decision to involve other should rest with the co‐producers themselves, that is, people with diagnosis of bipolar disorder in this case.

## 5 Discussion: Conceptualising Co‐Production With People With Bipolar Disorder

The consultation suggested that for people with lived experience, research could feel impersonal or potentially exploitative, even while being described as motivating and worthwhile. The LEAP members reflect that trust helps in delineating conditions under which research might safe or unsafe and inform decision making. The four themes collectively conceptualise co‐production as a relational process and partnership between the research team and broader community partners.

Trust was constructed as a central facilitator of this relationship, enabling meaningful participation, sustained engagement and reduced risk of further marginalisation [23]. This aligns with broader patient partnership literature, in which power imbalances, jargon and unclear expectations are common barriers, while respectful relationships and visible change within the community act as facilitators [22]. LEAP members further linked mistrust about research legitimacy with experiences associated with bipolar disorder itself. When research is perceived as impersonal, unverified or lacking in transparency, researchers risk compounding existing vulnerabilities rather than enabling participation. Therefore, third sector organisations or other service providers with established community relationships can bridge gaps between academic researchers and people within the bipolar disorder community [3, 24].

Similarly, a focus on trust should not oversimplify structural inequality. The consultation with LEAP members further iterates this. Even where relationships are positive, researchers typically retain control over research processes like funding, analysis, publication and career development. LEAP members advised on the study and process design; however, the interpretive authority remained with the academic team.

### 5.1 Consultation on the Engagement Spectrum

Using the IAP2 spectrum [11], this one‐off LEAP consultation is best understood as *consultation*, where researchers sought lived experience input to inform later co‐production design. However, it does not (on its own) meet the overarching NIHR INVOLVE definition of co‐production shared power and responsibility across a project [10]. This paper, therefore, aims to contribute to how to include people with bipolar disorder in designing co‐production processes. This consultative step is considered valuable, especially because it aims to inform collaborative practice in the next step.

### 5.2 Identity of Co‐Producers and Language

While not a primary analytic focus, the nature of co‐production as a research method was briefly questioned by LEAP members. They expressed confusion over terms such as ‘co‐producers’, and there was a consensus that ‘participants’ felt more accessible. This observation highlights how language intended to signal partnership may itself create barriers to engagement.

This difference further points to unresolved questions about power. Even within participatory frameworks, researchers often retain institutional control, while people with lived experience contribute expertise without equivalent recognition. This is consistent with critiques that participatory methods do not automatically resolve epistemic injustice or power imbalance [4, 5, 25]. Therefore, the recommendations aim to offer strategies supporting more meaningful engagement without dissolving structural tensions.

### 5.3 Importance of Communication and Accessible Information

Language and communication underpinned many of the findings and recommendations. LEAP members emphasised using plain language for recruitment materials, understanding consent processes and other communication. Recent work by Lang, King [26] highlights that language in health research could be inaccessible for people with lived experience, and it is important to translate complex terminology to have ongoing dialog with community partners [27]. Freebairn, Occhipinti [28] further state that person‐centred and jargon‐free language is important to create an inclusive environment in mental health research.

In the present consultation, LEAP members included frequency, sharing progress and accessibility as an important part of communication. This alludes that communication practices are important to maintain trust, safety and meaningful participation.

### 5.4 Connections to Existing Engagement Frameworks

Findings of the consultation closely align with community‐based participatory research and other existing engagement frameworks. Recruitment through trusted organisations and peer networks emphasise partnership with trusted organisations and existing networks. The emphasis on visible impact states that impact should be treated as important in establishing meaningful engagement [22]. Fair payment and expert recognition further align with evidence that inadequate compensation and unclear influence undermine partnership [13]. The present consultation complements previous literature by focusing on the design of advisory‐group recruitment and communication for bipolar disorder research.

### 5.5 Strengths

A key strength of this paper is the direct focus on the voices of people with bipolar disorder and the attempt to generate condition‐specific practical recommendations. The consultation produced actionable guidance that aims to inform both AMBIENT‐BD and future studies. The use of RTA [20] provided a systematic and theoretically explicit approach to developing themes from the data, relating to IAP2, INVOLVE and CBPR and other existing frameworks.

### 5.6 Limitations

Similarly, the limitations of this paper should be acknowledged. The small sample of LEAP members and the single consultation session mean that the findings represent initial reflections rather than a comprehensive account of the bipolar disorder community. The analysis prioritised the insights offered by LEAP members. This approach aligns with growing recognition that initial consultations with people with bipolar disorder can generate valuable guidance for more inclusive methodology [29].

LEAP members endorsed convenience sampling methods like word of mouth and existing supporting organisation. This might create an over‐reliance on the same people and under represent people who are less connected to these groups or networks. Over consulting same community partners can bias the perspectives available to researchers.

Additionally, conducting the consultation online was both an advantage and a disadvantage. It enabled inclusivity but may have limited sense of physical trust and important community‐building aspects. LEAP members were already engaged with AMBIENT‐BD study, which may have skewed reflections towards the value of lived experience involvement. Future work (especially next stage of the study) should seek advise from people who have not previously held advisory roles to get more critical perspectives. Finally, the single‐session design captures initial reflections but does not show how views might evolve through sustained engagement over time.

## 6 Conclusions

This paper was designed to give direction to the next stage of AMBIENT‐BD's co‐production work, where people with bipolar disorder will be recruited to identify clinical and functional priorities. Although based on a one‐off consultation, the study explored how people with bipolar disorder conceptualise co‐production by offering practical recommendations. The four themes‐ communicating with co‐producers, providing accessible information, maintaining trust and impact as a motivator, collectively frame co‐production as a relational process for meaningful engagement. The results and recommendations further offer provisional and bipolar disorder‐specific set of insights grounded in lived experience. This work aims to provide a foundation for more comprehensive frameworks that can guide future co‐production research in bipolar disorder and mental health conditions.

## Author Contributions

**Raahat Manrai:** conceptualisation, methodology, investigation, formal analysis, writing – original draft, data curations. **Amber Roguski:** methodology, writing – review and editing. **Amy Ferguson:** methodology, resources, writing – review and editing. **Iain Campbell:** methodology, resources, writing – review and editing. **Daniel J. Smith:** project administation, funding acquisition, writing – review and editing. **Aja L. Murray:** supervision, validation, writing – review and editing. **Maria Gardani:** supervision, validation, writing – review and editing.

## Conflicts of Interest

The authors declare no conflicts of interest.

## Supporting information


Supporting File

## Data Availability

Data can be made available upon request.
