# Supplementary material for: Developing Best Practices in Co‐Production With Lived Experience Experts in Bipolar Research: Insights From the AMBIENT‐BD Lived Experience Advisory Panel
Source: Health Expect. 2026 Oct 5;29(5):e70887. doi: 10.1111/hex.70887 (PMC13636650; doi:10.1111/hex.70887)
Supplement: Supplementary file 1 [file HEX-29-e70887-s001.docx]

# AMBIENT-BD Co-production Session Plan

## Introduction

Thank you for agreeing to take part in this co-production. Co-production means that you will help us and our research team co-design our research activities and processes going forward.

As a part of this session, we will be engaging in some discussions about how to involve people with bipolar disorder in the main AMBIENT-BD study. This one-off consultation session will help us bring more people with bipolar disorder on board and recruit a diverse set of people for the course of our study.

It is important to mention that we will be recording this session, however the recordings will not be shared with anyone even within the AMBIENT team. The recordings will be transcribed and all your data will be de-identified and anonymised before being analysed.

We have planned for this session to be for 45-60 minutes; however, we can change the duration of our upcoming sessions depending on how you feel. We will be having a small break during the session, but please feel free to call for a short break/timeout if you require. We will ask general questions about engaging people with bipolar disorder in future research.

It is important that we mention that we are not expecting you to share any of your personal experiences. However, in case you feel distressed or triggered by the information discussed in the group, please feel free to take a small break or let someone from the team know and they would be able to talk to you.

May I double-check that you are happy for this interview to be recorded?

[Continue recording, once the participants have confirmed their consent.]

**Before we formally start, let me do a quick 5-minute presentation explaining the process of co-production especially within the context of AMBIENT-BD.**

## Questions

We will be devised some themes to discuss in the sessions going forward. These questions are focus on engaging people with BD so you are not required to share any of your personal experiences.

- **Prior research experience-**
  - Have you taken part in any previous research projects before? What do you understand by them?
    - What had been your prior experience or understanding of research?
    - How can researchers reach out to people from hard-to-reach communities?
- **Providing information to co-producers-**
  - How to provide participant information to the co-producers? (For e.g., Having a video recording of the researcher giving out participant information for people to see the research team)
  - How can we communicate complex and potentially difficult information to people? How can researchers avoid making people feel distressed? What type of language to use?
  - How can we ensure that people understand the
- **Gathering consent from the co-producers-**
  - How to gather consent from our co-producers? How to ensure that people know what they are signing up to?
  - How to navigate days where people are not feeling well?
    - How should researchers contact people who are not feeling well?
    - Would it be considered appropriate to contact the person on a later date? If so, how many times would it be considered appropriate to contact a person?
- **Participant involvement- We need people with BD to be involved in different aspects of the study. This includes involvement within research design, participant recruitment and knowledge exchange and generating impact (as mentioned in the introductory presentation).**
  - As per your experience and knowledge, which aspects of the study do you think the co-producers are most likely to be involved in?
    - Research designing, testing the devices, having discussions around scientific research
    - How can we ensure that the co-producers are engaged throughout the entire process of research?
    - How can we create an environment where everyone is comfortable to share their views in the sessions?
- **Co-production sessions- Here we are trying to understand how to organise our different sessions.**
  - As per your understanding, what would be the best way to conduct our sessions?
    - Do you reckon having in-person or online sessions could have a better involvement or engagement with the co-producers? (Keeping in mind that LEAP members are global)
  - How should our first session look like?
  - We have planned the themes for our sessions to look like- Introduction, Discussion of Clinical Outcomes, Discussion of Functional Outcomes, Discussion around sleep devices – choosing volunteers for sleep devices, Testing the devices, Conversation around our research methodology- EMA prompts. How should we organise the order of our sessions?
  - How should we end the process of co-production? What should our last session look like?
  - How should we give feedback to our co-producers? How frequent should it be?
- **Decision making & group dynamics-**
  - How do we make decisions within our sessions? Should we use votes, views or ranking different priorities? How can we negotiate differences within people opinions?
  - How can we make sure that everyone’s voices and experiences are reflected?

Closing question

Is there anything else you would like to discuss that has not already been covered? I hope everyone feels settled and comfortable to call the session off. Thank you very much for taking part in this session. Please feel free to contact me in case of any questions.
